# Mechanical behavior of reservoir bank slope–pile–sheet structures under reservoir operation: Field monitoring and numerical simulation analysis

**DOI:** 10.1371/journal.pone.0339875

**Published:** 2026-01-02

**Authors:** Zhiwei Cai, Tongqing Wu, Lei Nie, Yue Wu, Zhao Xiang, Zhijie Yang, Nianchun Xu, Fei Qi

**Affiliations:** 1 CCTEG Chongqing Engineering (GROUP) Co., Ltd., Chongqing, China; 2 School of Civil and Hydraulic Engineering, Chongqing University of Science and Technology, Chongqing, China; 3 China Construction Eighth Engineering Division Co., Ltd., Shanghai, China; 4 Guang’an Vocational and Technical College, Guang’an, China; 5 Chongqing Youzhou Construction Co., Ltd., Chongqing, China; Guizhou University, CHINA

## Abstract

Long-term fluctuations in reservoir water levels can lead to the deterioration of bank slope materials, representing a key trigger of instability. This study investigated the behavior of a slope–pile–sheet support structure at a site in Chongqing’s “Two Rivers and Four Banks” area through an integrated program of field monitoring and numerical simulation. The results demonstrated a strong correlation between slope displacement/settlement and water-level fluctuations, exhibiting a characteristic three-stage process. Rapid drawdown triggered substantial horizontal displacement with a one-month response lag, while settlement primarily occurred during water-level rise. Earth pressure behind the piles exhibited a non-linear R-shaped distribution, with a delayed response in shallow layers and a pronounced local pressure drop at 8 m depth indicative of seepage erosion. The pile bending moment showed a distinct S-shaped profile, with a maximum positive moment (1978.44 kN·m) at the rock-soil interface (13 m) and a negative moment zone below 21 m. The bending moment response also exhibited a one-month lag and was particularly sensitive to rapid drawdown. The identified contraflexure point at 21 m depth provides a basis for pile length optimization. The close agreement between numerical simulations and field data validates the strong hydro-mechanical coupling in the system. This research provides theoretical and practical support for the design and optimization of similar support structures in reservoir bank environments.

## 1 Introduction

**Fig 1:**
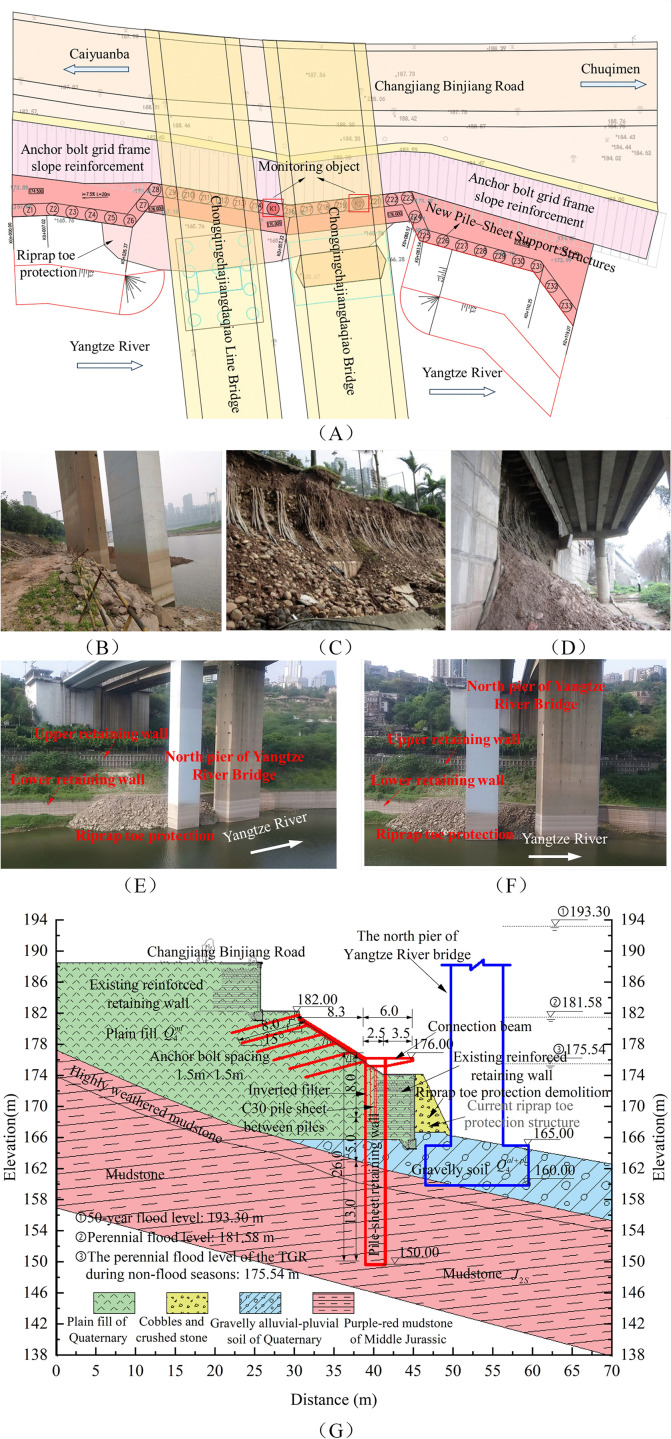
Site location and engineering conditions of the studied bank slope. **(A)** Location map of the project area along the Yangtze River in Chongqing. **(B-D)** Photographs showing typical failure modes of the existing reinforced soil retaining wall, including erosion of the backfill material, rupture of the reinforcing elements, and loosening of the panel connectors. **(E-F)** Emergency stabilization measures using rock dumping. **(G)** Geological cross-section of the slope after implementation of the pile-sheet reinforcement system.

Long-term periodic fluctuations in reservoir water levels significantly deteriorate the engineering properties of slope rock and soil, making this a key factor inducing slope instability. These fluctuations subject the soil and rock mass to repeated cycles of wetting-drying, soaking, and unloading, leading to the degradation of their physical and mechanical properties, particularly a gradual reduction in shear strength [1,2]. Studies have shown that even a 5% reduction in the shear strength of the rock and soil mass can notably lower the safety factor, demonstrating the high sensitivity of slope stability to changes in shear strength [3,4]. A prominent example is the Three Gorges Reservoir (TGR), where operational water-level variations between 145 m and 175 m have triggered numerous large-scale landslides and collapses [2,5,6]. These destabilizing effects are particularly pronounced during rapid water-level rises or falls, exhibiting significant hysteresis and cumulative damage characteristics that can ultimately lead to slope failure [7,8]. In Chongqing’s main urban area, the “Two Rivers and Four Banks” reinforced soil retaining walls, constructed primarily in the 1990s, have suffered severe deformations. These include cracks, bulging, and stair-step cracking, resulting from the combined impacts of extreme floods in 1998 and 2020, as well as persistent water-level fluctuations following the impoundment of the TGR. In some cases, these structures have even exhibited outward tilting and collapse (Fig 1). To address these issues and enhance the safety and serviceability of these reservoir bank structures, a comprehensive improvement project for the “Two Rivers and Four Banks” has been initiated, creating an urgent need for effective repair and reinforcement strategies.

Slope stability in reservoir areas is critically controlled by water-level fluctuations. To elucidate the associated deformation and failure mechanisms, researchers have employed diverse methods, including engineering geological analysis [9,10], limit equilibrium methods [1,11,12], numerical simulation [1,3,13–15], and physical modeling [7,16–19]. For instance, Tang et al. [9] characterized the structure and evolution of the Huangtupo landslide using a 3D multi-field monitoring system; Kamran et al. [15] combined monitoring with coupled seepage-stress modeling to assess the impacts of reservoir operation and rainfall; and Chen et al. [20,21], Hu [22], and Song et al. [23] correlated water-level changes with deformation and failure modes through field surveys and monitoring. Collectively, these studies establish hydrodynamic conditions as a dominant factor controlling reservoir landslide behavior.

The pile–sheet retaining wall is widely used for slope stabilization along high-speed railways [24,25], highways [26,27], and other engineering projects [28,29] due to its high overall stability, strong anti-sliding capacity, and flexible pile arrangement. Substantial research has focused on its reinforcement mechanisms and design, primarily using numerical simulation [24,25,30–36], theoretical analysis [37,38], and experimental studies [30–33,35,39–43]. However, a critical limitation is that these investigations have predominantly considered conventional loading conditions. For example, Xing et al. [25] and Ma et al. [33] investigated the mechanical behavior and stress characteristics of pile–sheet walls during and after excavation. Zhang et al. [37] developed a theoretical method for optimizing anti-slide pile spacing based on the soil arching effect, while Deng et al. [41,42] and Lian et al. [43] used shaking table tests to explore the seismic performance and dynamic responses of these structures. Although these findings form a crucial knowledge base, they do not adequately account for the intense hydrodynamic actions induced by periodic reservoir operation.

Furthermore, periodic reservoir water-level fluctuations degrade the geomechanical properties of bank slope materials. This degradation causes a dynamic redistribution of loads on the supporting structures, ultimately compromising their bearing capacity and long-term safety. To address this, researchers have recently employed physical model tests to investigate the performance of various support structures under water-level fluctuations or rapid drawdown [44–47]. While these laboratory studies provide valuable insights into structural behavior and failure mechanisms, they inherently simplify geological complexity, scale effects, and boundary constraints. Consequently, in-situ monitoring and field testing of pile–sheet structures remain essential for evaluating their long-term performance under actual reservoir operating conditions.

This study focuses on a typical bank slope reinforcement project along Changbin Road in Chongqing. We established a comprehensive field monitoring system for the slope–pile–sheet support structure to track displacement, settlement, earth pressure, bending moment, and shear force. Using data from a full hydrological year (February 2022 to March 2023) and a complementary numerical model, we analyzed the displacement and deformation of the structure under fluctuating reservoir water levels. We also investigated the variation patterns of earth pressure behind the piles and the bending moment of the piles. Furthermore, the stress characteristics of the pile–sheet structure under different reservoir operation conditions were examined. The study reveals the deformation and stress response mechanisms of the bank slope–pile–sheet support system under dynamic water-level changes. Based on these findings, we propose optimization recommendations, providing a theoretical basis and technical support for the design of similar engineering projects.

## 2 Site characterization and engineering background

### 2.1 Condition assessment of the existing retaining wall

The bank protection projects along the “Two Rivers and Four Banks” in Chongqing’s central urban area were primarily constructed in the 1990s. Reflecting the economic conditions and construction technologies of that era, mechanically stabilized earth (MSE) walls were utilized for slope protection in several sections. Most of these structures have now reached or exceeded their design service life. After prolonged exposure to a complex hydrogeological environment involving river immersion, flood scouring, and frequent water-level fluctuations, significant deterioration has occurred.

This study focuses on a representative section between Caiyuanba and Chuqimen along Changbin Road, adjacent to the northern bank of the Shibanpo Yangtze River Bridge (Fig 1A). The original structure was a typical MSE wall system consisting of concrete panels, tensile reinforcing elements, and compacted backfill. This section featured a two-tiered configuration: an upper wall 5.0 m high with 11 reinforcement layers spaced at 0.30 m intervals, and a lower wall 8.0 m high with 23 layers at the same spacing. A 1:1.5 sloped section, protected by hexagonal concrete blocks, connected the two walls. The backfill primarily consisted of plain fill, underlain by gravelly soil serving as the bearing stratum (Fig 1G).

The most prevalent distress mechanisms observed in these MSE walls primarily involve the erosion and scour of backfill material, the aging and fracture of polymeric or metallic reinforcements, and the corrosion or loss of integrity in panel connection systems.

These mechanisms have led to visible distress in the wall face, including cracking, bulging, and tilting. In critical areas, the development of cavities and localized collapses has been observed, significantly compromising the global stability of the structure. Furthermore, the seasonal “winter storage and summer release” operation of the TGR, which drives water-level fluctuations between 145 m and 175 m, has accelerated the degradation of the backfill soil’s shear strength, exacerbating the damage and triggering slope movements behind the walls (Fig 1B-1D). Although temporary stabilization measures, such as rock dumping at the toe, have been implemented (Fig 1E-1F), permanent reinforcement is urgently required to ensure the long-term safety of the bridge and surrounding infrastructure.

### 2.2 Geological and hydrological characteristics

The project site is located on the first terrace of the Yangtze River in Chongqing. The subsurface stratigraphy (Fig 1G) comprises, from top to bottom: (1) Quaternary plain fill (Q_4_^*ml*^), (2) Quaternary gravelly alluvial-pluvial deposits (Q_4_^*al+pl*^), and (3) bedrock of the Middle Jurassic Shaximiao Formation, consisting of purple-red mudstone (J_2_s).

**Fig 2:**
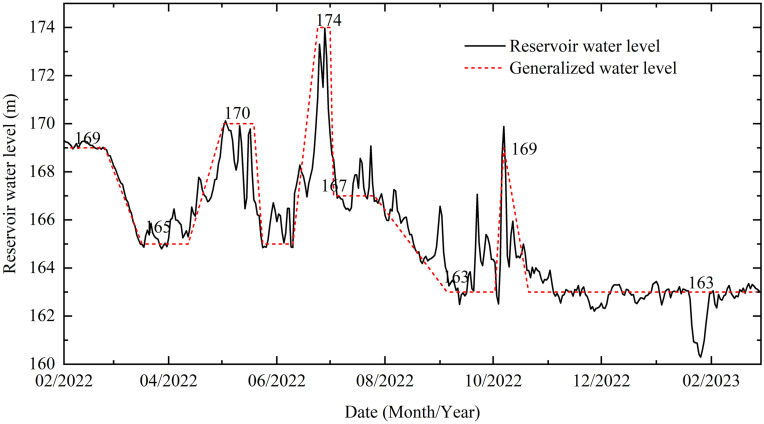
Reservoir water level time series and its generalized trend from February 2022 to March 2023.

The study area, situated in the upper reaches of the Yangtze River, is influenced by the TGR. The river channel is relatively narrow, exhibiting prominent gorge-like geomorphology. Water levels are governed by both regional precipitation and reservoir operations, resulting in substantial periodic fluctuations. A 30-meter vertical drawdown zone has been established within the reservoir area, which critically impacts bank slope stability and the performance of retaining structures. According to data from nearby monitoring stations, the historical maximum water level reached 193.30 m (recorded on August 20, 2020). The daily water level variation and its generalized trend from February 2022 to March 2023 are presented in Fig 2.

### 2.3 Implemented reinforcement scheme

A composite vertical-inclined slope reinforcement system was designed based on a comprehensive stability analysis of the original retaining walls and field investigation data, to ensure the safety of the Yangtze River Bridge piers and adjacent bank protection facilities. A typical cross-section of the reinforced slope is shown in Fig 1G.

The primary foundation element of the new system is a pile–sheet retaining wall constructed directly behind the original MSE wall. The wall comprises cast-in-place piles, each with a length of 26.0 m, a diameter of 2.5 m, and a center-to-center spacing of 4.0 m. The piles are rock-socketed to a depth of 13.0 m into the underlying mudstone. Precast concrete sheets (C30 grade), with a thickness of 0.3 m and dimensions of 1.5 m (length) × 1.0 m (width), are installed between the piles. Drainage holes are incorporated into the sheets to mitigate hydrostatic pressure behind the wall.

A cantilevered scenic walkway was constructed atop the pile–sheet wall at an elevation of 176.0 m, with a width of 6 m. In addition to its aesthetic function, the walkway provides a structural surcharge. The back slope was further reinforced with grouted soil anchors, each with a diameter of 25 mm, a length of 8.0 m, and an inclination of 15°. The anchors are arranged in a staggered pattern with horizontal and vertical spacings of 1.5 m. This integrated reinforcement scheme effectively enhances the overall stability and deformation resistance of the bank slope.

## 3 Materials and methods

A comprehensive on-site monitoring system was established to investigate the mechanical behavior of the pile–sheet structural system under reservoir operation conditions. This research was conducted within an active civil engineering project: the Emergency Remediation Project for the Retaining Wall at Shibanpo Yangtze River Bridge in Chongqing. Formal permissions for site access and data collection were granted by the project owner, the Chongqing Yuzhong District Binjiang Construction Management Office, and the construction contractor, Chongqing Water Conservancy Harbor and Channel Construction Group Company Limited, as stipulated in the construction contract and a research collaboration agreement.

As the monitoring activities were an integral and authorized component of the construction work and were confined to the project site, no separate approvals from external ethics committees or environmental authorities were required. All procedures were non-destructive and had no impact on the structural integrity or the surrounding environment.

The monitoring system was designed to track key mechanical parameters, including slope displacement, settlement at the pile tops, earth pressure behind the piles, and stress within the reinforcing steel bars. Data were collected manually at scheduled intervals to document the structural response to reservoir water level fluctuations. The resulting dataset provides essential validation for subsequent numerical simulations and safety assessments.

### 3.1 Experimental design and monitoring system setup

The field monitoring system comprised three main components: a displacement monitoring system, an earth pressure measurement system, and a rebar stress measurement system.

#### 3.1.1 Displacement monitoring.

**Fig 3:**
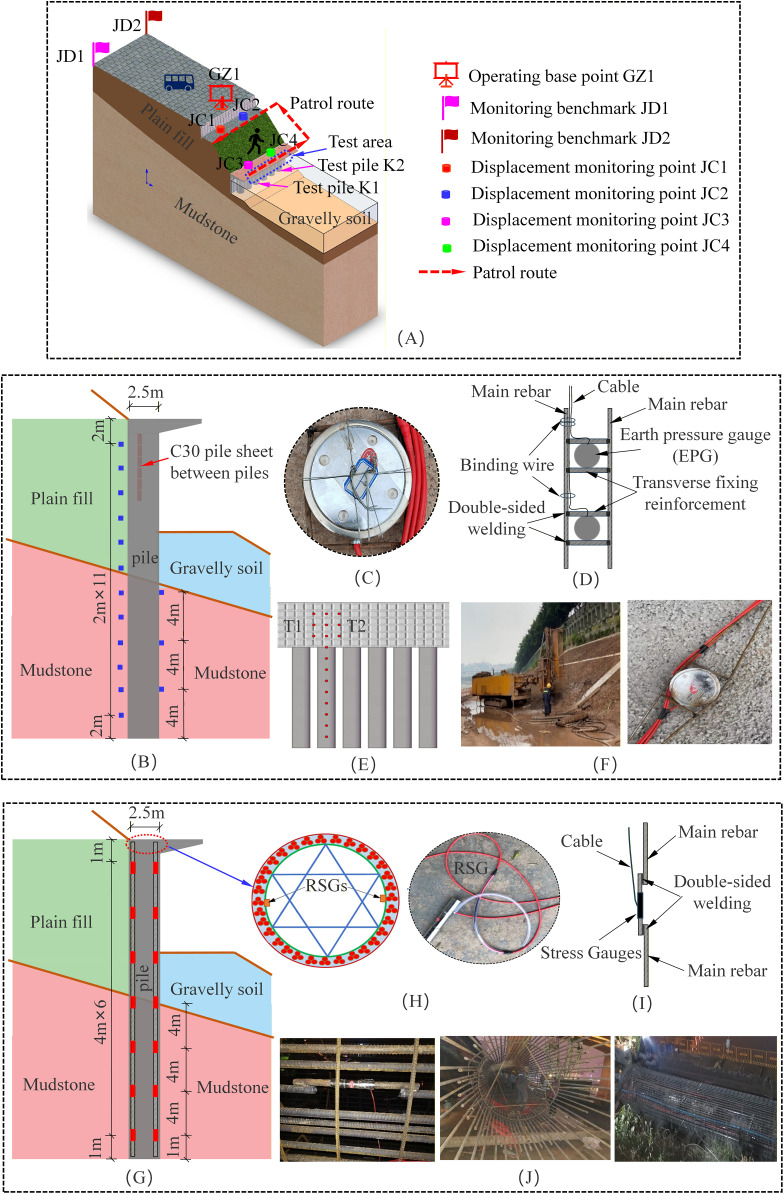
On-site monitoring system of pile–sheet structure. **(A)** Layout of surface displacement monitoring points. **(B)** Structural composition of an earth pressure gauge. **(C)** Installation method of an earth pressure gauge. **(D)** Profile layout of earth pressure gauges. **(E)** Schematic elevation layout of earth pressure gauges. **(F)** Field installation of earth pressure gauges. **(G)** Profile layout of rebar stress gauges along the pile depth. **(H)** Schematic diagram of rebar stress gauges in cross-section. **(I)** Schematic diagram of rebar stress gauge installation method. **(J)** Field installation of rebar stress gauges.

To investigate the displacement characteristics of the riverbank slope and pile tops over time, test piles K1 and K2 near the Yangtze River Bridge piers were instrumented for monitoring. In accordance with relevant monitoring standards [48], points JC3 and JC4 were established at the pile tops (elevation 176.00 m). Corresponding points JC1 and JC2 were installed at the slope crest (elevation 183.96 m). A reference point (GZ1) was set at the top of the upper retaining wall of Changbin Road (elevation 188.42 m). Benchmark points JD1 and JD2 (elevation 188.12 m), established during the reinforcement construction, were also used as references. All displacement measurements were conducted using a high-precision Topcon MS05AX2 total station. The layout of the displacement monitoring points is shown in Fig 3A.

#### 3.1.2 Arrangement of earth pressure gauges.

The layout of earth pressure gauges along the pile depth is shown in Fig 3B. To accurately capture the distribution of earth pressure behind the piles and retaining sheets, vibrating wire sensors were deployed on the soil-facing sides of test piles K1 and K2 at 2 m vertical intervals, and at 4 m intervals on the external side of the embedded pile segment. Similarly, gauges were installed behind retaining panels T1 and T2 at 2 m vertical intervals (Fig 3E). The gauges were installed using a “post-construction embedding” technique developed by the research team; the structural details and installation method are shown in Fig 3C and 3D, respectively, and field installation is documented in Fig 3F. This technique ensured the stability and accuracy of the monitoring data.

#### 3.1.3 Arrangement of rebar stress gauges.

The layout of rebar stress gauges along the pile depth is shown in Fig 3G. To measure rebar axial stress and analyze the stress characteristics of the pile–sheet system, 14 rebar stress gauges were uniformly installed along the longitudinal rebars of test piles K1 and K2. Starting from 1.0 m above the pile toe, the gauges were spaced at 4.0 m vertical intervals; their cross-sectional arrangement is illustrated in Fig 3H. The installation method, which employed double-sided welding, is detailed in Fig 3I, and field practices are shown in Fig 3J. Monitoring the longitudinal rebar stress enabled the determination of the internal force distribution within the pile, which facilitated the analysis of shear force and bending moment variations along the pile depth.

### 3.2 Monitoring schedule and data acquisition

In accordance with relevant standards [49,50], the monitoring duration was set to cover at least one hydrological year, with a data collection interval of one month or less. Monitoring frequency was increased during flood and rainy seasons. Specifically, for the pile–sheet structure, routine monitoring was conducted every 15–20 days, while during the flood season (June to September), monitoring was performed weekly. Bank slope displacement was observed every two months. All monitoring was carried out through periodic manual inspections and manual data collection to ensure data stability and traceability.

### 3.3 Results and analysis of on-site monitoring

To investigate the deformation characteristics and load-bearing mechanisms of a slope–pile–sheet structure under dynamic reservoir water level fluctuations, this study systematically processed and analyzed monitoring data collected over a complete hydrological year from February 2022 to March 2023. By processing raw data on slope displacement, pile top settlement, rebar stress, and earth pressure, key structural response parameters were derived. These parameters, including displacement patterns, earth pressure distribution, pile body bending moment, and shear force, were then analyzed to elucidate the structural behavior in response to reservoir water level changes.

#### 3.3.1 Displacement and deformation characteristics of the slope–pile–sheet system.

**Fig 4:**
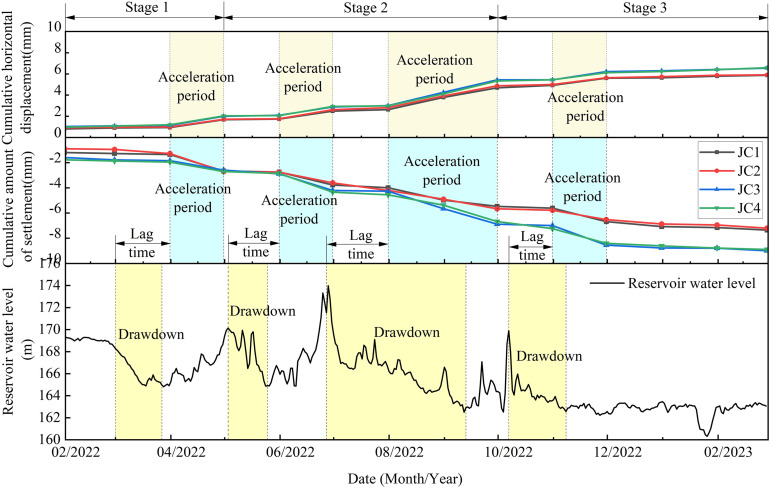
Response of pile head displacement and slope settlement to reservoir water level fluctuations.

Displacement and settlement data from monitoring points JC1–JC4 collected between February 2022 and March 2023 were analyzed in relation to simultaneous reservoir water level variations. Response curves illustrating the relationship between cumulative horizontal displacement and settlement with water level fluctuations are presented in Fig 4.

Based on reservoir operating conditions, the deformation process during the observation period was divided into three stages. In Stage 1 (High Water Level Operation, approximately 169 m), cumulative horizontal displacement and settlement increased slowly, indicating a stabilizing effect. In Stage 2 (Reservoir Water Level Rapid Drawdown), a rapid drop in water level led to a significant increase in both the magnitude and rate of deformation. In Stage 3 (Low Water Level Operation, approximately 163 m), the deformation rate slowed again, entering a relatively stable state.

As shown in Fig 4, displacement and settlement trends at all monitoring points strongly correlated with reservoir water level fluctuations. Cumulative settlement demonstrated higher sensitivity, closely tracking the water level, whereas cumulative horizontal displacement exhibited a response delay of approximately one month. This hysteresis is attributable to the low permeability of the fill material, which retards the internal pore water pressure response.

From June 19 to July 7, 2022, as the water level rose at 0.506 m/d, monitoring point JC4 exhibited the highest cumulative horizontal displacement rate (0.0287 mm/d). Subsequently, from July 7 to July 21, during a water level drop at 0.542 m/d, JC3 recorded the highest rate (0.0420 mm/d). These results indicate that a rapid decline in water level significantly accelerates horizontal displacement, while a rising level suppresses it. This is attributed to stabilizing hydrostatic pressure during rises and lagged groundwater drainage during drawdown, which increases effective soil stress.

Furthermore, front-edge points (JC3, JC4) generally experienced slightly higher cumulative displacement and rates than rear-edge points (JC1, JC2), due to more direct exposure to reservoir-induced groundwater fluctuations.

Settlement was also significantly impacted. During the June 19-July 7 rise, JC4 recorded the highest settlement rate (0.0497 mm/d). During the July 7–21 drawdown, JC2 exhibited the highest rate (0.020 mm/d). Overall, settlement increased more rapidly during water level rise, with rear-edge points showing greater settlement, likely due to prolonged saturation weakening soil cohesion and particle migration toward the front edge during drawdown creating voids.

#### 3.3.2 Earth pressure distribution in the pile–sheet structure.

**Fig 5:**
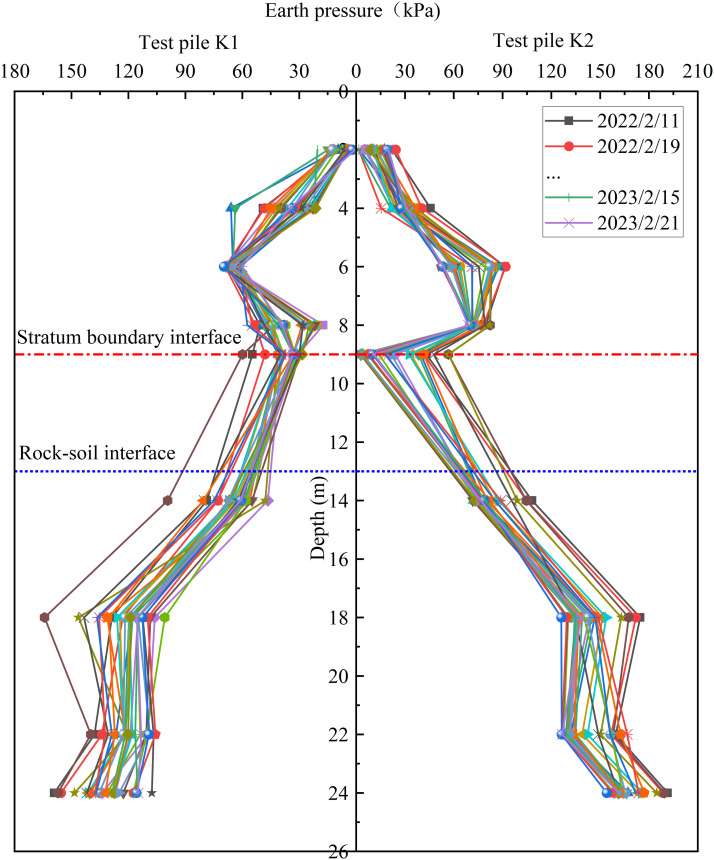
Earth pressure distribution behind test piles K1 and K2.

Earth pressure distribution behind test piles K1 (left) and K2 (right) at different depths is presented in Fig 5.

The earth pressure behind the pile generally exhibits an “R-shaped” distribution. Within the shallow depth range (0–9 m), pressure increased to maxima of approximately 69.68 kPa (K1) and 91.85 kPa (K2) at around 6 m depth, then decreased. A significant pressure reduction averaging 39.19% occurred near 8 m depth; for example, at pile K1, pressure dropped sharply from 66.62 kPa to 17.30 kPa. This abrupt decrease is attributed to seepage-induced soil erosion and particle migration during rapid reservoir drawdown, potentially leading to localized void formation. In the deeper section (9–24 m), earth pressure generally increased with depth, following a near-triangular distribution, with a minor reduction around 22 m depth.

Earth pressure variations were more pronounced above the stratum boundary, while changes below remained minor, indicating limited displacement and weak interaction between the overlying fill and the pile. The observed pressure reductions are likely due to soil particle erosion by groundwater flow during significant drawdown.

**Fig 6:**
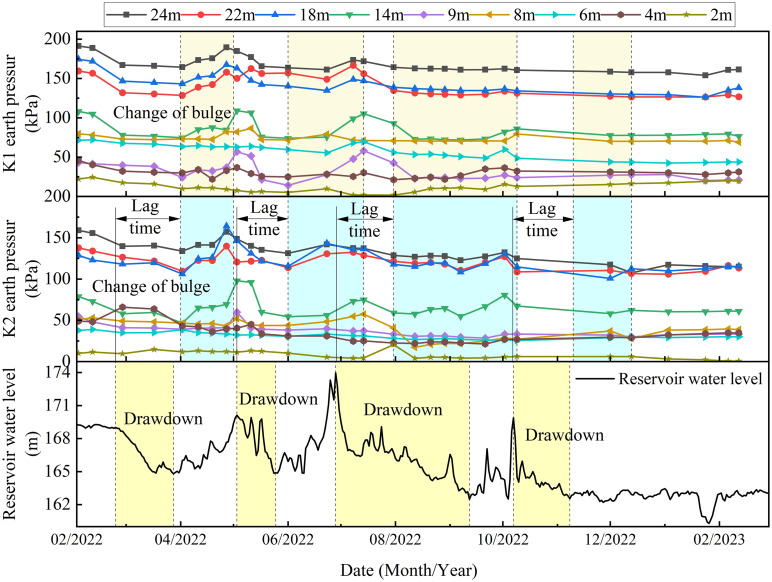
Temporal evolution of earth pressure at different points on test piles K1 and K2 during reservoir water level fluctuations.

The time-series evolution of lateral earth pressure throughout the hydrological year is shown in Fig 6.

Lateral earth pressures at depths from 2 m to 14 m showed limited variability, generally following near-linear trends. Changes below 9 m were especially minor, potentially due to imperfect gauge-soil contact, sensor misalignment, or soil particle loss. Notably, discernible variations were observed at 8 m and 9 m, correlating with reservoir level changes, though magnitudes remained small.

In contrast, at greater depths (14–24 m), a strong correlation was observed between lateral earth pressure and reservoir water level for both piles. Pronounced pressure changes during rapid drawdown or refill suggest a rapid hydraulic response, indicating pore water pressures in deeper soils are tightly coupled with reservoir fluctuations. Earth pressure gauges at shallower depths exhibited a delayed response, likely due to reduced permeability and slower hydraulic pressure transmission.

Furthermore, lateral earth pressures behind pile K1 at 9 m and behind pile K2 at 8–9 m peaked during the reservoir recession phase. This delayed peak mirrors horizontal displacement trends, indicating that earth pressures at greater depths respond more promptly and synchronously to hydraulic variations. This response pattern underscores the significant role of hydrostatic loading and the delayed hydraulic response of the upper soil profile.

#### 3.3.3 Distribution of pile bending moments in the pile–sheet structure.

**Fig 7:**
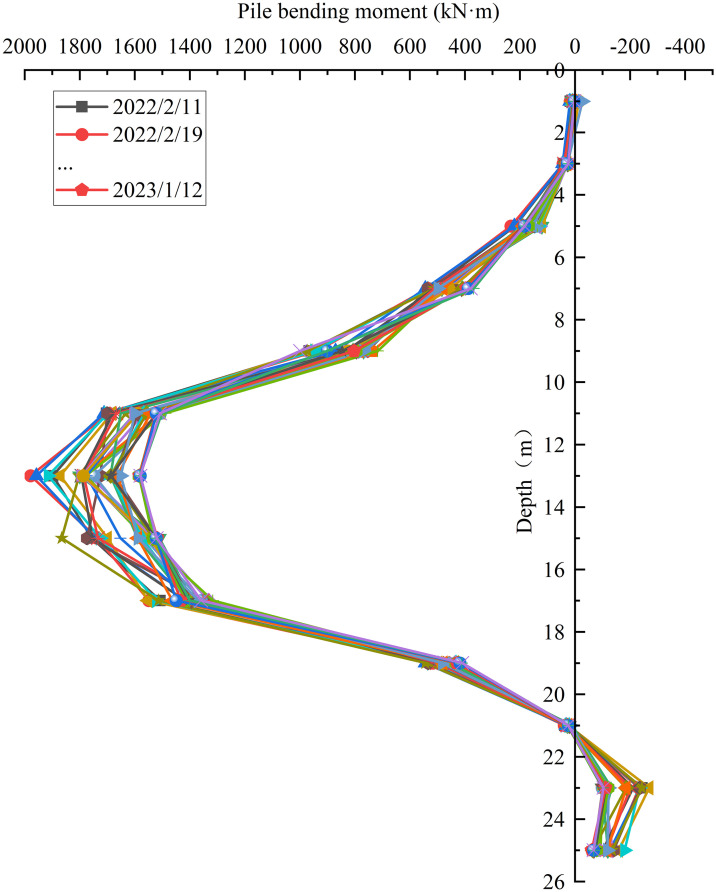
Bending moment distribution along the pile depth.

Bending moments of the test piles were determined from measured rebar stresses, with depth-wise distributions presented in Fig 7.

The bending moment distribution exhibits a characteristic S-shaped profile, indicating a transition from positive to negative moments along the pile depth. The maximum positive moment of 1978.44 kN·m occurs at the rock-soil interface (13 m depth), indicating significant stress concentration. Below this, the moment gradually decreases and reaches a point of contraflexure (zero moment) at approximately 21 m. A negative moment zone is observed between 21 m and 24 m depths, with a minimum of −179.93 kN·m, suggesting reverse bending potentially due to deep rock rebound or stress redistribution. Although minor fluctuations occurred, the overall distribution remained consistent, indicating relatively stable mechanical behavior.

**Fig 8:**
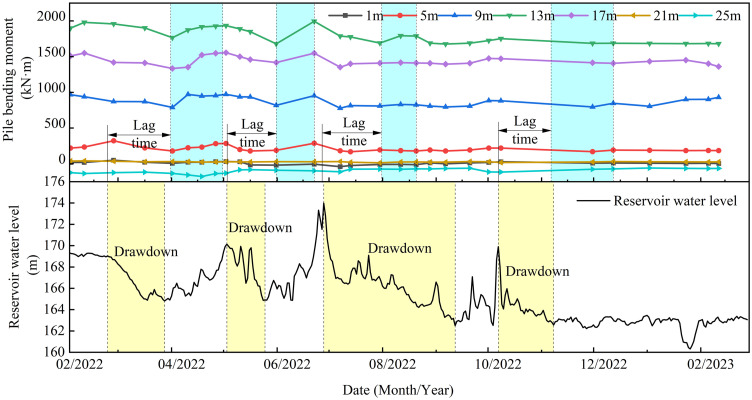
Temporal variation of bending moments in response to reservoir water level fluctuations.

The temporal variations of bending moments in response to reservoir water level fluctuations are shown in Fig 8.

As illustrated in Fig 8, the variation curves of pile bending moment and reservoir water level show an apparent correlation. However, due to the low permeability of the slope, pore water pressure transmission is significantly delayed, leading to a lag in the bending moment response. This is particularly pronounced near the embedded rock section at 13 m depth.

For example, from March 4 to April 6, 2022, the water level decreased at 0.128 m/d. The bending moment at 13 m depth subsequently increased by 9.52% between April 11 and May 12, 2022. Similarly, from May 12 to June 4, 2022, the water level dropped at 0.228 m/d. The bending moment subsequently increased by 18.79% between June 10 and July 2, 2022. These data indicate a bending moment response lag of approximately one month, with a higher rate of water level decline associated with a more significant moment increase.

Notably, during low water level periods (e.g., October 2022 to February 2023), bending moment fluctuations at most depths diminished significantly. For instance, at 5 m depth, the moment remained within ±27 kN·m, with a variation rate of less than 14%, indicating mechanical stabilization during dry seasons.

In conclusion, the pile bending moment demonstrates marked sensitivity to reservoir water level variations, exhibiting pronounced responses during rapid drawdowns and relative stability during low water level periods.

## 4 Discussion

### 4.1 Numerical simulation of the slope–pile–sheet structure

To further investigate the mechanical response of the slope–pile–sheet structure under reservoir operation conditions, a typical cross-section was selected, as shown in Fig 1G. Based on the project’s authoritative geotechnical investigation report and engineering practice, the physical and mechanical parameters of the slope geomaterials and pile–sheet structural elements were determined (see Table 1).

**Table 1:** Physical and mechanical parameters of the geomaterials and structural elements.

Classification	Unit cell type	Unit Weight *γ*/kN/m³		Internal friction angle *φ*/°		Cohesion force *c*/kPa		Young’s modulus *E*/MPa	Poisson’s ratio *μ*	Hydraulic conductivity K/ cm/s	Subgrade bearing capacity *f*_*a*_/kPa
		Natural	Saturated	Natural	Saturated	Natural	Saturated				
Plain fill	3D solid elements	21.7	21.9	28	25	10	8	3500	0.45	0.03	150
Gravelly soil		21.8	22	33	29	0	0	25	/	0.013	400
Moderately weathered mudstone		25	25.5	38		300		1.35 × 10^6^	0.38	5.85 × 10^−7^	2030
MSE		21	21.5	30		10		3500	0.45	0.03	/
Bridge and pile-sheet structure		26		/		/		3 × 10^7^	0.2	/	/
Anchor rod	1D embedded truss elements	78.5		/		/		1.96 × 10^8^	0.28	/	/

**Fig 9:**
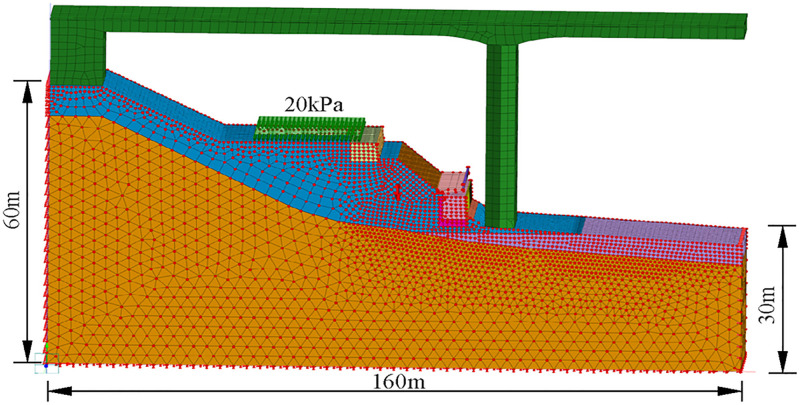
Numerical model of the slope–pile–sheet structure system.

A three-dimensional finite element model of the slope–pile–sheet system was developed using Midas/GTS software. The model employed a hybrid mesh of tetrahedral and hexahedral elements, comprising 175,962 elements and 125,106 nodes in total. A surface load of 20 kPa was applied at the top of the secondary retaining wall to represent pavement loading, as illustrated in Fig 9.

The developed model was then imported into FLAC^3D^ for further numerical calculation and analysis. The Mohr–Coulomb failure criterion was adopted to model the behavior of the soil and rock masses, while the pile and sheet structures were treated as linear elastic elements.

### 4.2 Comparative analysis between field tests and numerical simulation

To better analyze the stress response of the slope-pile-sheet support system under fluctuating reservoir water levels, pile K1, which was fully instrumented for field monitoring, was selected as the focus of this investigation. Based on comprehensive in situ measurements, a representative reservoir operating condition with the water level at 169 m was chosen for comparative evaluation of key structural parameters. These parameters include pile bending moment, shear force distribution, and earth pressure development behind the pile. This comparison aimed to provide deeper insights into the internal force transmission mechanisms and load-bearing behavior of the support system under fluctuating reservoir water levels.

#### 4.2.1 Comparison of pile bending moments.

**Fig 10:**
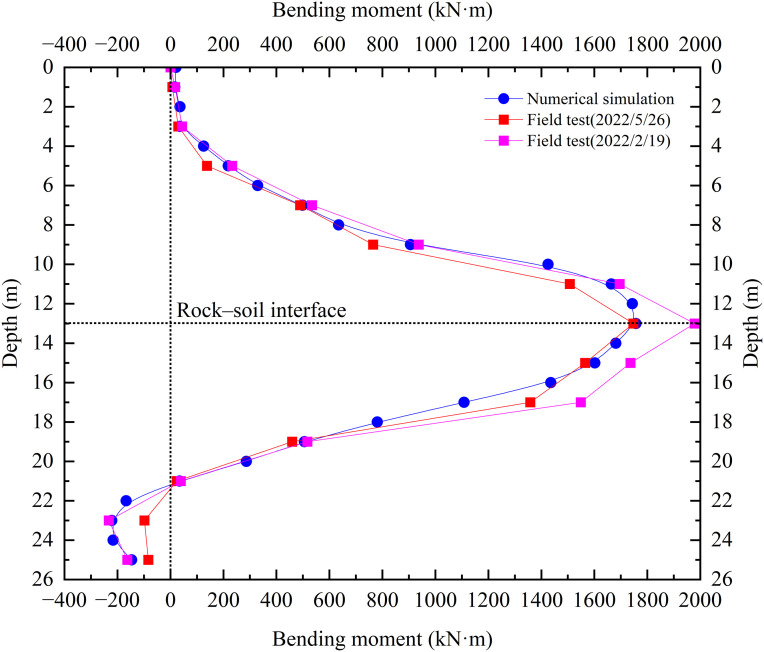
Comparative diagram of pile bending moment distribution under the 169 m water level condition.

A comparison between the measured and numerically simulated bending moment distributions along pile K1 under a 169 m reservoir water level is illustrated in Fig 10.

Both datasets exhibit a consistent S-shaped profile. Above the rock–soil interface, the bending moment increases with depth, reaching a maximum positive value near the interface. Below this zone, the moment progressively decreases and transitions into a negative region. The maximum bending moments from on-site measurements were 1978.44 kN·m and 1747.33 kN·m, compared to the simulated value of 1757.49 kN·m. The difference between measured and simulated values thus ranged from 0.6% to 12.6%, with all maxima occurring near the rock–soil interface. The contraflexure point (zero moment) was consistently identified at approximately 21 m depth in both results, suggesting potential for future pile length optimization.

#### 4.2.2 Comparison of pile shear forces.

**Fig 11:**
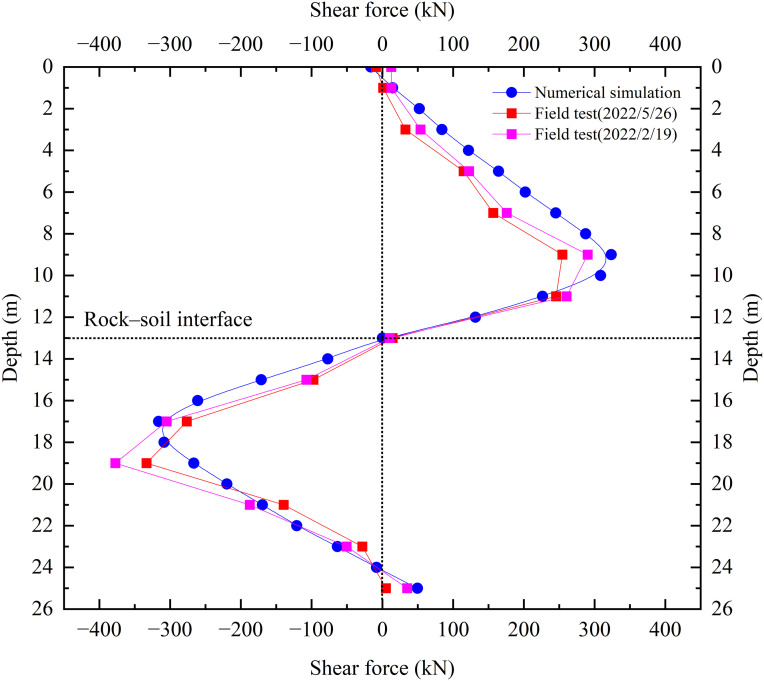
Comparative diagram of pile shear force distribution under the 169 m water level condition.

The distribution of shear force along pile K1 under a 169 m reservoir water level is presented in Fig 11.

The maximum shear forces from on-site measurements were 290.25 kN and 254.47 kN, both located at a depth of 9 meters. The numerical simulation predicted a higher maximum of 323.64 kN. Despite this difference in magnitude, both the measured and simulated results exhibit a consistent S-shaped distribution. The shear force direction is opposite on the upstream and downstream sides (positive downstream, negative upstream). Along the pile depth, the shear force magnitude initially increases and subsequently decreases.

Notably, the shear force reversal points coincide with the locations of bending moment extrema and contraflexure points. The first reversal point corresponds to the rock–soil interface, and the second is near the pile toe (approximately 23 m deep), highlighting the coupled interaction between shear and bending responses.

#### 4.2.3 Comparison of earth pressure behind the pile.

**Fig 12:**
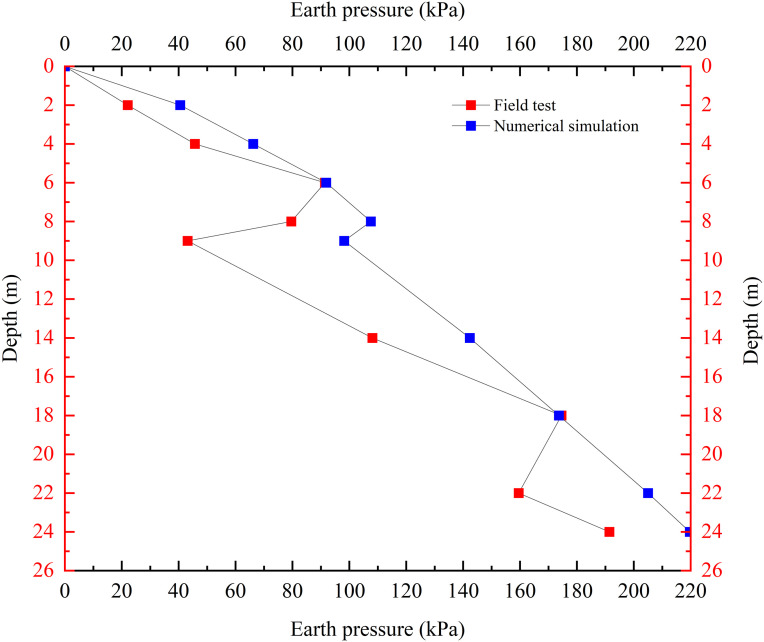
Comparative diagram of earth pressure distribution behind pile K1 under the 169 m water level condition.

The distribution of earth pressure behind pile K1 from both measurement and numerical simulation under a 169 m reservoir water level is presented in Fig 12.

Both datasets exhibit a general “R-shaped” distribution, characterized by an initial increase and subsequent slight decrease in earth pressure within the cantilevered portion, followed by an approximately linear increase in the embedded portion. A notable deviation is a local pressure drop in the field data at 8 m depth. This anomaly may indicate a discontinuity in stress transfer, potentially caused by soil erosion and particle migration during rapid reservoir drawdown—a mechanism not captured by the simulation. In contrast, the numerical model produces a more idealized, approximately triangular pressure distribution.

While the simulation does not fully replicate this local anomaly, the overall trend is consistent. This suggests that the model accurately reflects the general earth pressure behavior.

In summary, the numerical simulation provides a reliable prediction of the macroscopic earth pressure distribution. Despite local discrepancies, it offers significant value for the engineering analysis and design of pile–sheet support system.

## 5. Conclusions

This study investigated the behavior of a slope–pile–sheet support system under fluctuating reservoir water levels through an integrated program of field monitoring and numerical simulation. The key findings are as follows:

Displacement and settlement of the slope–pile–sheet system were strongly correlated with reservoir water-level fluctuations, exhibiting a clear three-stage response. In Stage 1 (high water level, approximately 169 m), both displacement and settlement increased slowly, indicating a stabilizing effect. In Stage 2 (rapid drawdown), deformation magnitude and rate increased significantly. In Stage 3 (low water level, approximately 163 m), the system re-entered a relatively stable state with slowed deformation rates. Rapid drawdown triggered substantial increases in horizontal displacement (e.g., 0.0420 mm/d at JC3), with a response lag of approximately one month due to the low permeability of the fill. In contrast, settlement primarily occurred during water-level rise (e.g., 0.0497 mm/d at JC4). Front-edge points exhibited higher displacement rates, resulting from more direct exposure to reservoir-induced groundwater fluctuations. These findings underscore the critical influence of hydrostatic pressure and pore water pressure dynamics on slope stability.

The earth pressure distribution behind the piles exhibited a non-linear R-shaped profile. A significant local pressure reduction, averaging 39.19%, was observed at a depth of approximately 8 m, indicating potential void formation from seepage-induced soil erosion during rapid drawdown. Earth pressures at depths of 14–24 m correlated strongly with reservoir level changes, responding rapidly to hydraulic variations, while shallower layers (2–14 m) exhibited a minimal or delayed response. Peak lateral pressures at 9 m (K1) and 8–9 m (K2) during water-level recession phases aligned with displacement trends, confirming the prompt hydraulic response in deeper soils. These results highlight the significant influence of hydrostatic loading and pore pressure transmission dynamics on the pile–sheet system.

Field monitoring and numerical simulations revealed a stable S-shaped bending moment distribution in the piles. The maximum positive bending moment (1978.44 kN·m) occurred at the rock-soil interface (13 m depth), with a negative moment zone (minimum of −179.93 kN·m) developing below 21 m. The bending moment response exhibited significant sensitivity to water-level fluctuations, with a lag of approximately one month. A higher rate of water-level decline correlated with a more pronounced increase in bending moment (e.g., an 18.79% increase at a drawdown rate of 0.228 m/d). The identified contraflexure point at 21 m depth suggests that the pile length could be optimized to approximately 23 m, providing a basis for more economical design. The close agreement between numerical results and field data validates the monitoring data’s reliability and demonstrates strong hydro-mechanical coupling in the pile–sheet structural response.

Collectively, these results provide theoretical and technical support for the design and optimization of similar slope–pile–sheet support structures in reservoir bank environments.
