# Correction: Mechanical behavior of reservoir bank slope–pile–sheet structures under reservoir operation: Field monitoring and numerical simulation analysis

**DOI:** 10.1371/journal.pone.0342195

**Published:** 2026-02-02

**Authors:** The PLOS One Staff

There are errors in the author affiliations. The correct affiliations are as follows:

Zhiwei Cai^2^, Tongqing Wu^1^, Lei Nie^3^, Yue Wu^1^, Zhao Xiang^4^, Zhijie Yang^3^, Nianchun Xu^1^, Fei Qi^5^

1 School of Civil and Hydraulic Engineering, Chongqing University of Science and Technology, Chongqing, China, 2 CCTEG Chongqing Engineering (GROUP) Co., Ltd., Chongqing, China, 3 China Construction Eighth Engineering Division Co., Ltd., Shanghai, China, 4 Guang’an Vocational & Technical College, Guang’an, China, 5 Chongqing Youzhou Construction Co., Ltd., Chongqing, China

The publisher apologizes for the error.
